# Molecular dynamics exploration of poration and leaking caused by Kalata B1 in HIV-infected cell membrane compared to host and HIV membranes

**DOI:** 10.1038/s41598-017-03745-2

**Published:** 2017-06-15

**Authors:** Wanapinun Nawae, Supa Hannongbua, Marasri Ruengjitchatchawalya

**Affiliations:** 1 Pilot Plant Development and Training Institution, King Mongkut’s University of Technology Thonburi (Bang Khun Thian Campus), 49 Soi Thian Thale 25, Bang Khun Thian Chai Thale Rd., Tha Kham, Bang Khun Thian, Bangkok 10150 Thailand; 2 Department of Chemistry, Kasetsart University, 50 Phaholyothin Rd., Ladyao, Chatuchak, Bangkok Thailand 10900; 3 Biotechnology Program, School of Bioresources and Technology, King Mongkut’s University of Technology Thonburi (Bang KhunThian Campus), 49 Soi Thian Thale 25, Bang Khun Thian Chai Thale Rd., Tha Kham, Bang Khun Thian, Bangkok 10150 Thailand; 4 Bioinformatics and Systems Biology Program, King Mongkut’s University of Technology Thonburi (Bang Khun Thian Campus), 49 Soi Thian Thale 25, Bang Khun Thian Chai Thale Rd., Tha Kham, Bang Khun Thian, Bangkok 10150 Thailand

## Abstract

The membrane disruption activities of kalata B1 (kB1) were investigated using molecular dynamics simulations with membrane models. The models were constructed to mimic the lipid microdomain formation in membranes of HIV particle, HIV-infected cell, and host cell. The differences in the lipid ratios of these membranes caused the formation of liquid ordered (lo) domains of different sizes, which affected the binding and activity of kB1. Stronger kB1 disruptive activity was observed for the membrane with small sized lo domain. Our results show that kB1 causes membrane leaking without bilayer penetration. The membrane poration mechanism involved in the disorganization of the lo domain and in cholesterol inter-leaflet translocation is described. This study enhances our understanding of the membrane activity of kB1, which may be useful for designing novel and potentially therapeutic peptides based on the kB1 framework.

## Introduction

Kalata B1 (kB1), a stable cyclic peptide in the cyclotide family^1, 2^, contains 29 amino acid residues, including six conserved cysteine residues that divide the amino acid sequence into six regions, referred to as loop 1 to loop 6^1, 2^. kB1 has potential uses in various applications, from agriculture^3, 4^ to therapeutics^5–7^. The bioactivities of the cyclic peptide arise via a common mechanism that is related to its ability to bind and disrupt the cell membrane^8^. The mechanism is initiated by the absorption of kB1 molecules onto the membrane surface^9, 10^. As it is energetically unfavorable for kB1 to penetrate the membrane^11^, kB1 prefers the area referred to as the membrane interfacial zone^10, 11^, which includes atoms at the membrane-water interface and the atoms of lipid hydrocarbon chains^12^. Aggregation of kB1 in this zone induces a membrane perturbation where lipids are lifted above the bilayer plane^10^, which leads to lipid extraction at high kB1 concentration^10, 13^. The peptide ultimately engenders leakage in the cell membrane via the poration process^9, 14^. Interestingly, kB1 does not require specific protein receptors to initiate its membrane activity^15, 16^; however, it exhibits different membrane disruption levels for different lipid compositions^8, 17^. The impact of the lipid properties on the membrane binding and activity of kB1 has received increased attention^17–19^. Phospholipids with phosphatidylethanolamine (PE) polar groups have been reported to promote an interaction between kB1 and the membrane and to enhance the disrupting activity of the peptide^14, 16–19^. In addition to PE lipid, cholesterol and sphingolipid also impact the membrane disrupting activity of kB1^13, 17^. However, their roles in the membrane response to the binding of kB1 remain unclear. Cholesterol decreases the fluidity of membrane lipids, especially saturated lipid, whereas sphingolipid contains saturated acyl chains^20^. The preferential interaction between these two lipids results in the formation of a lipid microdomain known as the raft domain, which is phase separated from the non-raft domain^20, 21^. The latter domain is enriched with unsaturated lipids. Therefore, the properties of a bilayer in the lipid microdomain are different from the non-microdomain^22, 23^. The microdomain has been reported to be involved in several cell activities, including signal transduction^24^, disease development^20^, and HIV infection^20, 25^. HIV recognizes a protein receptor that is localized in the lipid microdomain of the host cell membrane and initiates its invasion^20, 25, 26^. To generate new particles, the virus uses the host cell membrane as an envelope. Therefore, the lipid compositions of HIV particle, HIV-infected cell, and host cell membranes are similar^27–30^. However, the budding of HIV is specific to the lipid microdomain and is cholesterol dependent^25, 31^. The amounts of cholesterol and sphingomyelin have been reported to be significantly increased in the viral membrane compared to the HIV-infected cell and host cell membranes^27–30^. In relation to the anti-HIV activity of kB1, a series of studies has indicated that the cytoprotective concentration of kB1 against HIV infection is less than the concentration that is cytotoxic to the host cell^6–8^. The differences in the ratios of cholesterol and sphingolipid between the membranes may affect the disrupting activity of kB1^8^.

We previously applied a molecular dynamics (MD) simulation method to investigate the membrane activity of kB1^10, 11^. The studies used a Martini coarse-grained (CG) force field^32–34^ to simulate interactions between molecules. This force field has been successfully used to investigate the formation of the lipid domain in membrane models that contain diC16:0-PC (dipalmitoyl-phosphatidylcholine, DPPC), diC18:2-PC (dilinoleoyl-phosphatidylcholine, DLiPC), and cholesterol (CHOL) lipids^23, 35^. The membrane models contain the same lipid composition has been used to investigate the interactions between the lipid microdomain and membrane proteins^36–38^. Because membranes of HIV particle, HIV-infected cell, and host cell contain different amounts of cholesterol and sphingomyelin^28^, the size of the microdomains in these three membranes might be different. The aim of this study is to investigate the interplay between the binding of kB1 and the formation of differentially sized lipid microdomains. The membrane models contained DPPC, DLiPC and CHOL lipids. DPPC was used to represent sphingomyelin in the real membranes, while DLiPC and CHOL were used to represent all unsaturated lipids and cholesterol, respectively. Our simulations were divided into two conditions. In the first condition, simulations were conducted to investigate the effect of kB1 binding on the properties of the membranes, while the simulations in the second condition were conducted to investigate the implications of the membrane properties on the binding of kB1. In the latter condition, the simulations were conducted using the effective concentration of kB1^10^.

## Results

### Different properties of the viral membrane from compared to the host and infected cell membranes

**Figure 1:**
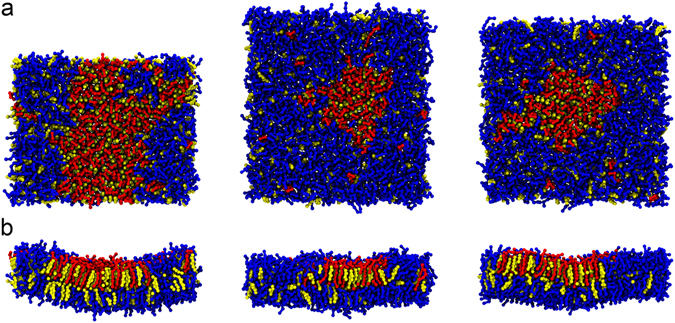
Membrane models. The (**a**) top and (**b**) side views of the VI, IN and HO membrane models are shown from left to right. DPPC, DLiPC and CHOL are shown as CPK models with red, blue and yellow colors, respectively.

In this study, membrane models, named VI, IN, and HO, were constructed to mimic the formation of lipid microdomains in membranes of HIV particle, HIV-infected and uninfected host cells, respectively. The simulation of each membrane, which contained 1,000 lipid molecules, was conducted for 20 µs (Supplementary Table S1). The DPPC:DLiPC:CHOL ratios of the VI, IN, and HO membranes were 0.13:0.38:0.49, 0.06:0.67:0.28, and 0.07:0.62:0.31, respectively. We calculated the ratios using the lipid compositions reported by Aloia *et al*.^28^ In the outer layer of each membrane, a DPPC/CHOL-rich domain was laterally separated from the DLiPC-rich domain (Fig. 1A). Using the density-based spatial clustering of applications with noise (DBSCAN) algorithm^39^, the ratios of DPPC, DLiPC and CHOL in the DPPC/CHOL rich domains of these membranes were 0.54:0.04:0.42, 0.59:0.10:0.32 and 0.55:0.07:0.37, respectively. The amounts of DPPC and CHOL in the domain of the VI membrane were 111 ± 7 and 86 ± 9 molecules greater than in the HO (59 ± 2 and 40 ± 5) and IN (43 ± 5 and 23 ± 4) membranes, respectively. The bilayer in the DPPC/CHOL-rich domain was approximately 0.4–0.5 nm thicker than the DLiPC-rich domain. These findings are consistent with a study of the phase separation of the liquid ordered (lo) domain from the liquid disordered (ld) domain in membrane models that contained the same lipid composition^23^. The DPPC/CHOL-rich and DLiPC-rich domains were identified as the lo and ld domains that presented as the lipid microdomain and the non-microdomain, respectively^23^. The sizes of the lo domains in the different membranes sorted in descending order are VI ≫ HO > IN.

The compactness of each membrane was determined as the area per lipid head bead (APL) in which a lower APL reflects a higher compactness. The APLs averaged over all of the lipid molecules in the VI, IN, and HO membranes were 0.48 ± 0.08, 0.62 ± 0.06 and 0.60 ± 0.07 nm^2^, respectively. For all of the membranes, the average APL in the outer layers was approximately 8–15% lower than the inner layer (Supplementary Fig. S1). More specifically, the average APL of the molecules in the lo domain was lower compared to the ld domain. Inversely proportional to the size of the lo domain, the surface area of the VI membranes (638.8 ± 6.8 nm^2^) was approximately 13 and 16% smaller than the HO and IN membranes (783.3 ± 7.8 and 816.9 ± 7.2 nm^2^), respectively. Therefore, the surface areas in ascending order are VI < HO < IN. These findings indicate that the differences in the lipid ratios cause the formation of lo domains of different sizes, which affects the compactness and surface area of the membrane models.

A curved surface was observed in the area of the lo domain for all membrane models (Fig. 1B). The radius of the curved surface in the VI membrane (approximately 1.18 nm) was the smallest, while the radii for the IN and OH membrane were nearly equal (approximately 1.98 and 1.93 nm, respectively) (Supplementary Fig. S2). The position of each lipid molecule in every layer of each membrane was analyzed by measuring the distance amplitude along the membrane normal axis (see the Methods section). The average amplitude was the highest in the VI membrane, followed by the HO and IN membranes, respectively. The amplitudes in the outer layer of these membranes were approximately 28, 60, and 40% lower than the inner layer, respectively (Table 1). We also observed that the lo domain contained lipids with lower amplitudes than the lipids in the ld domain (Fig. 1B). Correspondingly, the lipid tilt angle analysis (Supplementary Fig. S3) revealed that the PC lipids in the ld domain tilted toward the lo domain center; i.e., the lipid molecules from peripheral of the lo domain orient their head group to the center. The tilt angles of lipid molecules at the center of the domain are lower than the surrounding lipid molecules (Supplementary Fig. S4).

**Table 1:** Development of membrane perturbation induced by the binding of kB1.

	Membrane type	Level of the distance amplitudes	The outer layer							The inner layer						
			Time (µs)							Time (µs)						
			0	20	40	60	80	100	100*	0	20	40	60	80	100	100*
Amplitude (nm)	**VI membrane**	**1**	0.81	0.98	1.31	1.35	1.54	1.57	1.63	1.12	1.31	1.62	1.67	1.79	1.72	1.79
		**2**	NA	3.86	4.02	4.17	4.09	4.20	4.07	NA	3.44	3.8	3.49	3.85	3.90	4.08
		**3**	NA	NA	6.37	6.65	7.14	7.26	6.99	NA	NA	NA	NA	NA	6.18	6.11
	**IN membrane**	**1**	0.42	1.40	1.40	1.60	1.65	1.74	1.48	1.03	1.58	1.53	1.69	1.75	1.89	1.55
		**2**	NA	3.25	3.97	4.03	4.33	4.47	4.48	NA	3.37	3.54	4.02	4.19	4.37	4.34
		**3**	NA	NA	6.25	6.62	7.06	7.33	7.33	NA	NA	NA	NA	6.30	6.72	7.54
	**HO membrane**	**1**	0.66	0.58	0.55	1.02	1.35	1.50	1.40	1.10	1.23	0.74	1.23	1.55	1.56	1.54
		**2**	NA	NA	NA	NA	3.53	3.74	4.50	NA	NA	NA	NA	3.32	3.64	4.50
		**3**	NA	NA	NA	NA	NA	NA	6.71	NA	NA	NA	NA	NA	NA	6.35
Number of lipid (% to total)	**VI membrane**	**1**	100	95	78	74	56	50	58	100	95	74	68	57	56	49
		**2**	0	5	19	23	32	38	38	0	5	26	32	43	43	50
		**3**	0	0	3	4	11	12	4	0	0	0	0	0	1	1
	**IN membrane**	**1**	100	95	81	62	50	46	56	100	87	85	62	55	50	60
		**2**	0	5	19	35	38	40	25	0	13	15	38	40	40	28
		**3**	0	0	1	3	10	15	16	0	0	0	0	5	10	22
	**HO membrane**	**1**	100	100	100	100	94	78	36	100	100	100	100	98	80	30
		**2**	0	0	0	0	6	22	42	0	0	0	0	2	20	25
		**3**	0	0	0	0	0	0	6	0	0	0	0	0	0	13

*The distance amplitudes are measured using the second simulation condition.

NA: not applicable.

### Sensitivity of the membrane models to kB1 binding

The previous results indicate that the differences in the lipid ratios resulted in the formation of differentially sized lo domains, and that this also affected the properties of the membrane models. In the first simulation condition, MD simulations were conducted to investigate how each membrane model changes in response to the binding of kB1. Similar to our previous study^10^, the concentration of kB1 in each system was increased stepwise every 20 µs (see the Methods section). This protocol was applied in this study for its convenience of experimentally regulating the number of kB1 molecules that bind to each membrane. In each concentration step, the membrane system was continued to the next concentration step when the number of kB1 molecules bound to it was almost equal to the number of kB1 in the other two membranes. As a result, the amounts of kB1 molecules that bound to the VI, IN, or HO membranes were approximately equal (Table 2). In the following four subsections, we describe the differences in the response levels between the membrane models when they were bound to approximately equal numbers of kB1 molecules.

**Table 2:** Amount of kB1 molecules that bound to each membrane model.

Time (µs)	20	40	60	80	100	100*
Concentration step	24 kB1	48 kB1	72 kB1	96 kB1	120 kB1	350 kB1
VI membrane	20	41	61	79	85	83
IN membrane	23	43	65	82	87	100
HO membrane	18	41	60	77	84	90

*These values were measured using the second simulation condition.

### Membrane binding pattern of kB1

**Figure 2:**
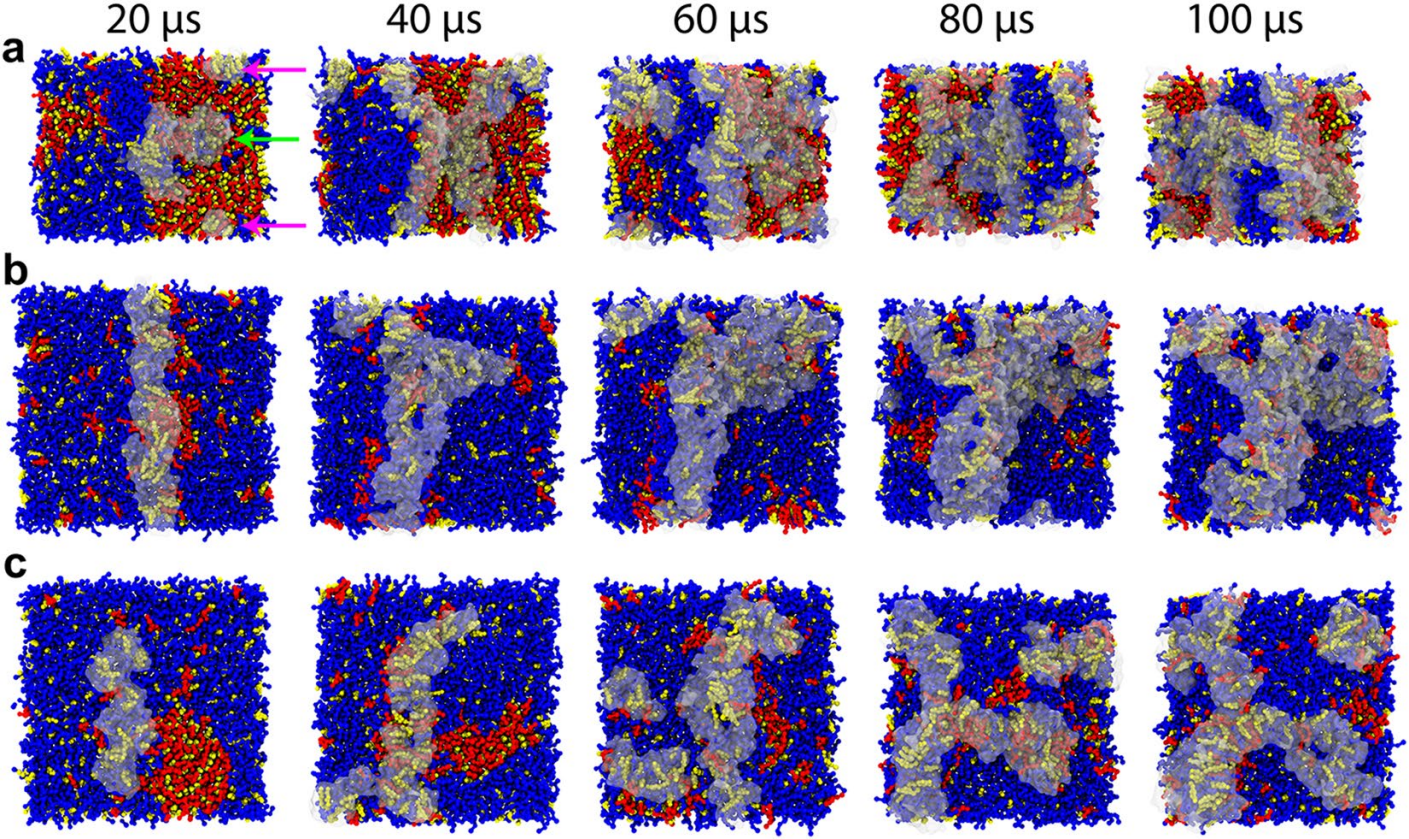
CHOL clustering and disorganization in the lo domain caused by binding of kB1. The binding of kB1 molecules with the (**a**) VI, (**b**) IN, and (**c**) HO membranes are shown from the top view of each system. Only kB1 molecules that directly interact with the membranes are shown, i.e., those with transparent surface models. DPPC, DLiPC and CHOL are shown as CPK models with red, blue, and yellow colors, respectively. The small and large oligomers that initially bind to the lo domain of the VI membrane are indicated with pink and green arrows, respectively. Please note that there is only one small oligomer, which is split by a periodic boundary.

The results in Fig. 2 show that molecules of kB1 bound only to the outer layer of each membrane that contained a lo domain. They formed oligomers in the water and subsequently bound to the membrane to form tower-like and wall-like oligomers. For the VI membrane, most of the kB1 molecules bound to the interface between the lo and ld domains. However, there were two kB1 oligomers that bound to the lo domain. The first oligomer was small and isolated. Within 20 µs, this oligomer moved laterally from the lo domain to the lipid domain interface (pink arrow in Fig. 2). In contrast, the second oligomer was a tower-like oligomer in which molecules at the bottom of the tower bound to the lo domain and molecules at the top bound with other kB1 molecules located at the lipid domain interface (green arrow in Fig. 2). The molecules that bound to the lo domain remained at their position throughout the simulation. Nevertheless, there was DLiPC lateral movement from the ld domain to these kB1 molecules. For the IN and HO membranes, all of the kB1 molecules that bound to the membrane occupied the ld domain, and they frequently moved laterally to the domain interface. These molecules were never observed to move into the area of the lo domain. Importantly, the binding of kB1 to different areas, in relation to the lipid domain, occurred by chance in each membrane, which indicates that different simulation runs can result in different binding locations of kB1 on the membrane. However, our results from all of the membrane models suggest that kB1 molecules tend to be located in the ld domain. This observation is also in accordance with our previous results^11^, which showed that monomeric and tetrameric forms of kB1 disfavor the lo domain.

### Disorganization of the lo domain

We observed clustering of CHOL with the kB1 molecules that bound to each membrane (Fig. 2). The size of the CHOL cluster increased when the amount of kB1 molecules that bound to the membrane increased (Supplementary Fig. S5). After 100 µs, the amount of CHOL molecules located within 1.2 nm of the kB1 molecules that bound to the IN membrane, which accounted for 30% of the total CHOL molecules in this membrane, was slightly higher than that in the VI (29%) and HO (25%) membranes. Moreover, the amount of CHOL molecules in the outer layer of each membrane increased, whereas it decreased in the inner layer (Supplementary Table S2). For the VI, IN, and HO membranes, the number of CHOL molecules in their outer layers increased by approximately 39, 52, and 23%, respectively. These findings imply that the binding of kB1 induced the translocation of CHOL from the inner layer to the outer layer, and that this binding is more likely to occur in the IN membrane than in the VI and HO membranes. Approximately 40% of these translocated molecules were clustered with the membrane-bound kB1 molecules. Additionally, Fig. 2 shows that the higher the number of kB1 molecules bound with each membrane, the smaller size of the lo domain. For the VI membrane (Fig. 2a), the initial lo domain (the domain at 0 µs) was observed to be separated into smaller domains after 20 µs. The size of the largest separated domain at 100 µs was approximately 31% (68 ± 4 DPPC and 67 ± 6 CHOL molecules) smaller than the initial domain. In the IN membrane (Fig. 2b), a clear cluster of DPPC and CHOL was not detected from 60–100 µs when most of the CHOL molecules in the outer layer were clustered with the membrane-bound kB1. For the HO membrane (Fig. 2c), the total amount of lipid molecules in the lo domain at 20 and 40 µs accounted for 78 (48 ± 2 DPPC and 29 ± 4 CHOL molecules) and 51% (33 ± 8 DPPC and 17 ± 5 CHOL molecules) of the amount at 0 µs, respectively. At 100 µs, a clear cluster of DPPC and CHOL was not observed. These results suggested that binding of kB1 induced the lateral disorganization of the lo domain. This process was observed to relate to the clustering of CHOL with kB1 and, hence, to depend on the size of the lo domain and the number of kB1 molecules that bind to each membrane.

### Progressive perturbation development in the different membranes

**Figure 3:**
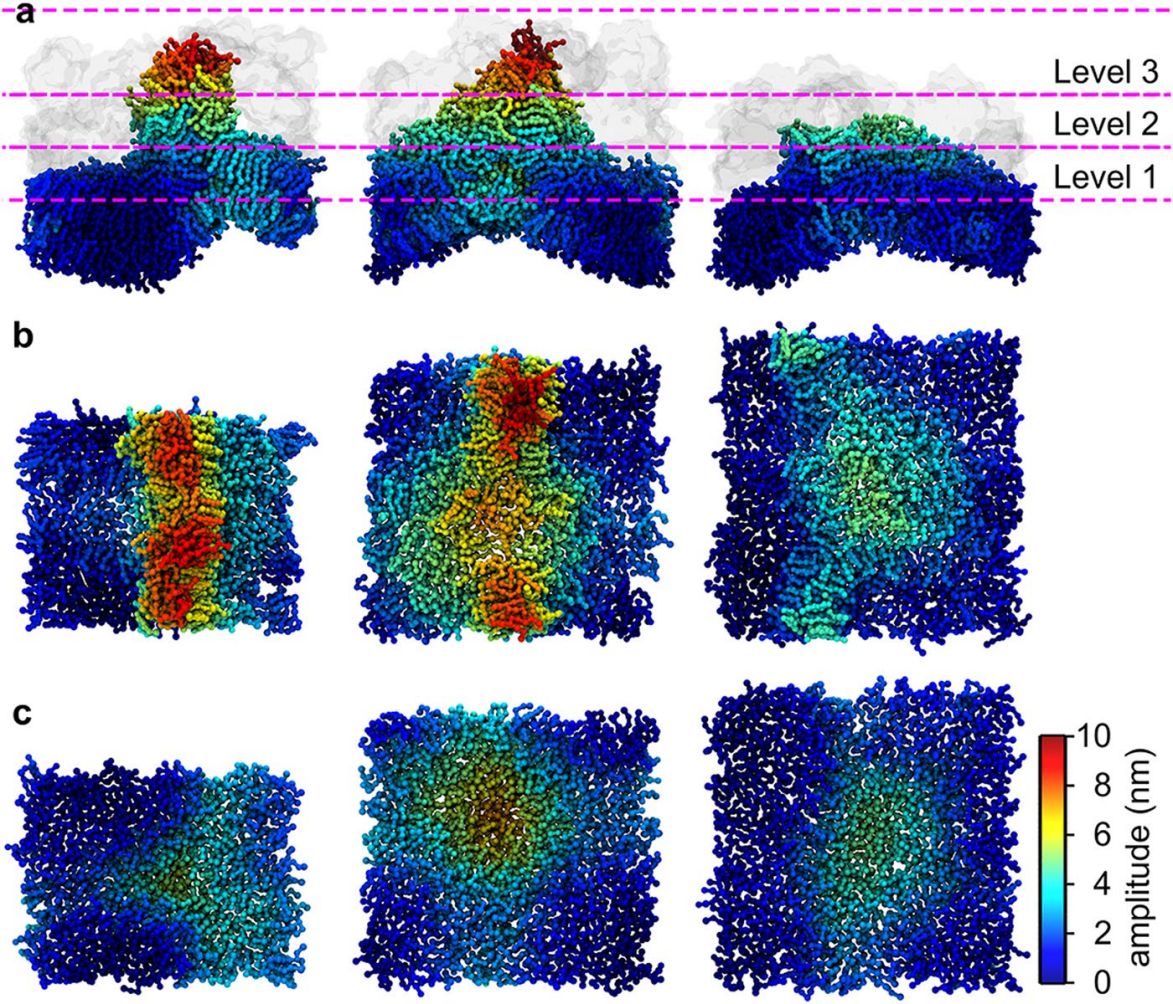
Levels of membrane perturbation. (**a**) Side views of the VI, IN, and HO membranes at 100 µs are shown from left to right. Clusters of kB1 molecules are shown as transparent surface models. The dashed lines show the levels of the distance amplitude in the outer layers of the membranes. The (**b**) top and (**c**) bottom views of the outer and inner layers of the membranes are shown. The lipid molecules are shown as CPK models. The color of each lipid molecule represents its amplitude (see the color scale bar). In (**b**) and (**c**), the kB1 molecules are not shown.

The binding of kB1 has been reported to induce membrane perturbation where lipid molecules are lifted from their original positions^10^. The degree of perturbation was quantified by calculating the amplitudes (as described above). Fig. 3 shows the perturbation at 100 µs of all the membranes used in the first simulation condition. The perturbation was classified into three different levels based on their amplitudes: lipid molecules were defined as the first, second, or third level if their amplitudes were in a range of 0.0–3.0, 3.1–6.0 or greater than 6.0 nm, respectively. These levels reflect the base, main, and peak levels of the amplitudes (Fig. 3a). Table 1 compares the development of progressive perturbation in each layer of the membranes. At 0 µs, 100% of the lipids in both layers were classified in the first level for all membranes. The amplitudes subsequently increased when the number of kB1 molecules that bound to each membrane increased. In the outer layer, most lipids in the second and third levels were lifted onto the ring-like hollow of kB1 on the membrane surface^10^. The tilt angles of the PC lipids in these levels were in a range of 60–90° (Supplementary Fig. S6), and they were localized in a narrow area across the membrane surface, indicating a monolayer folding in the outer layer. Based on the tilt angles, the amount of PC lipids in the folding area of the VI, IN, and HO membranes at 100 µs accounted for 30, 38, and 20%, respectively, of the total PC molecules in the outer layer of each membrane. For the VI membrane, the monolayer folding was only identified in the ld domain with flanking by small pieces of the lo domain (Fig. 3). In VI membrane, lipids in the second and third levels were identified for the first time at 20 and 40 µs, respectively. Approximately 38 and 12% of the total lipid molecules at 100 µs were classified in the second and third levels, respectively (Table 1). The same progression of the perturbation development was identified in the outer layer of the IN membrane with slightly higher amplitudes than those of the VI membrane (approximately 40 and 15% of the total lipids were identified in the second and third levels, respectively). The perturbation in the HO membrane was lower than other two membranes (approximately 22% of the total lipids were identified in the second level and there were no lipids in the third level). Therefore, the perturbation levels of the membranes in descending order are IN > VI ≫ HO.

For the inner layers, the lipids did not cluster in a narrow area as demonstrated in the outer layer, and only 5, 14, and 8% were identified to have tilt angles between 60–90° in the VI, IN, and HO membranes, respectively. The results indicate that folding did not occur in the inner layer. Instead, the lipids in the third level of this layer were identified as a small peak on the top of the perturbed surface (Fig. 3c). On the XY plane of the membrane, this peak was located at a position that corresponded to the edge of the ring-like hollow (Fig. 3b and c). For all of the membranes, the amplitudes and amounts of the lipid molecules in the second and third levels of the amplitudes in the inner layer were lower than the outer layer (Table 1). These findings imply that perturbation in the inner layer is a consequence of perturbation in the outer layer.

### Changes in lipid membrane compactness

In all membranes, the average compactness increased (APLs decreased) when the amount of kB1 that bound to the membrane increased (Supplementary Fig. S7). The results show that the compactness of the lipids in the outer layer increased at a higher rate than the inner layer (the APLs were 50% lower at 100 µs). The APLs in the VI, IN, and HO membranes decreased by approximately 66, 61, and 46%, respectively. Lipids with very low APLs were identified in the folding area (Supplementary Fig. S8a). In the inner layer, the APLs decreased by 38, 41, and 20%, respectively. The lipid molecules in the HO membrane, which exhibited lower distance amplitudes than the VI membrane, had higher APLs. However, the APLs in the IN membrane were higher than the VI membrane (Supplementary Fig. S5), which is most likely because the amount of CHOL in the inner layer of this membrane is lower than the VI membrane (Supplementary Table S2). Some lipids in the peak area of the IN membrane exhibited relatively higher APLs than the surrounding molecules (Supplementary Fig. S8b). Overall, these findings indicate that the IN membrane was the most sensitive to the binding of kB1, followed sequentially by the VI and HO membranes.

### Different membranes have different amounts of bound kB1

In the second simulation condition, we investigated the effect of the membrane properties on kB1 binding. Different from the first condition, the binding of kB1 was not controlled in this condition. Table 1 shows that the amounts of kB1 molecules that bound to the various membranes were different. The number of kB1 molecules that bound to the VI membrane, which has the smallest surface area, was 17 and 7 molecules lower than the IN and HO membranes, respectively. The number in this condition was also slightly lower than that in the first condition. There were no kB1 molecules that bound to the lo domain. As a result, the size of the lo domain at 100 µs (72 ± 7 DPPC and 78 ± 9 CHOL molecules) was larger than it was at the same time in the previous condition. The tilt angle analysis indicated that most of the PC lipids in the ld domain tilted toward the center of the lo domain where lipids were classified in the first level of the amplitudes (Table 1). The monolayer folding in the outer layer predominantly occurred in the ld domain, which occupied a small area of the VI membrane. Thus, the number of lipid molecules classified in the third level was approximately 67% lower than the previous condition, and the average amplitude was reduced by 4%. These findings indicate that maintenance of the lo domain limited the perturbation in the outer layer of the VI membrane. In the inner layer, the amplitude was very similar to the results identified in the previous condition. For the HO membrane at 100 µs, the number of membrane-bound kB1 was 6 molecules higher than that identified in the first condition (Table 2). The development of perturbation in the third level was first identified in this simulation condition, and the average amplitudes for both layers was greater than the previous condition. The amplitudes were as high as those identified in the VI membrane (Table 1). For the IN membrane, which had the largest surface area, the number of membrane-bound kB1 molecules and the average amplitudes were significantly higher than the other membranes (Tables 1 and 2). Compared to the previous condition, the average amplitudes in the outer layer did not change. However, in the inner layer, the amplitude was approximately 0.82 nm higher. These findings indicate that the surface area of the membrane, which was inversely correlated to size of the lo domain, regulated the number of kB1 molecules that bound to the membrane. Therefore, different ratios of the lipids, which regulated the domain size, could indirectly regulate the perturbation level caused by the binding of kB1.

### Poration in the IN membrane model

**Figure 4:**
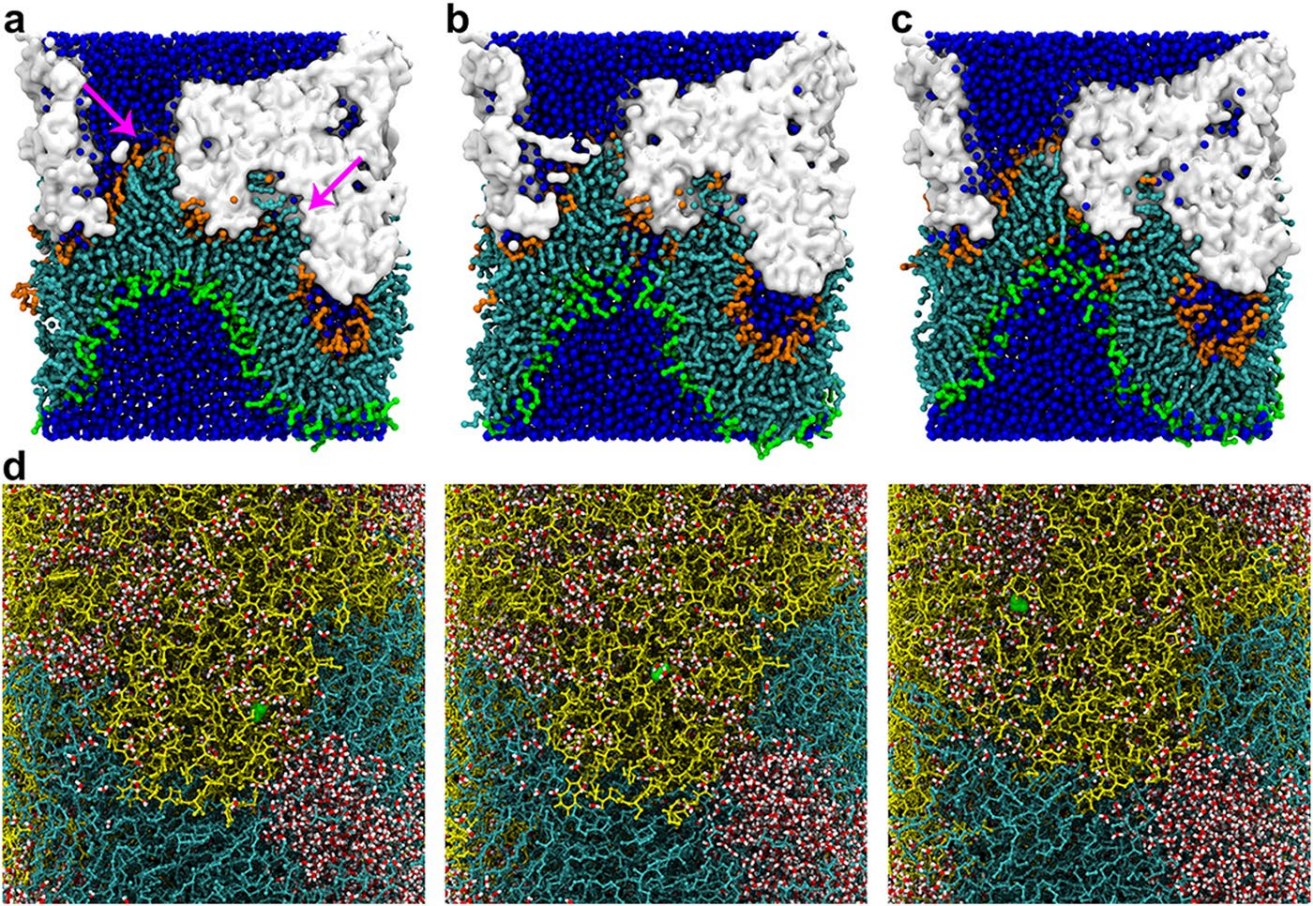
Process of membrane poration and water leaking. (**a–c**) Side views of the CG model of the IN membrane during the formation of a membrane pore are shown. The lipid extraction, water permeation and monolayer fusion, and pore expansion are shown sequentially from left to right. To detail the membrane changes during the poration mechanism, only the area of the simulation box that covers the membrane and the KB1 cluster are shown for each step. The lipid molecules are shown as CPK models in cyan. The head groups of the lipid molecules in the outer and inner layers are shown in orange and green, respectively. The water beads are shown as blue balls. A cluster of kB1 molecules is shown via a surface model in white. The arrows indicate two clusters of lipid molecules that are extracted from the membrane. (**d**) Side view of the AA model of the same membrane. The lipid molecules are shown as licorice models in cyan. The water molecules are shown via CPK models with white and red representing the hydrogen and oxygen atoms, respectively. The kB1 molecules in the cluster are depicted as licorice models in yellow. A water molecule represented by a green space-filling model is shown as an example of a water molecule that is leaking from the inner layer to the outer layer of the membrane.

**Figure 5:**
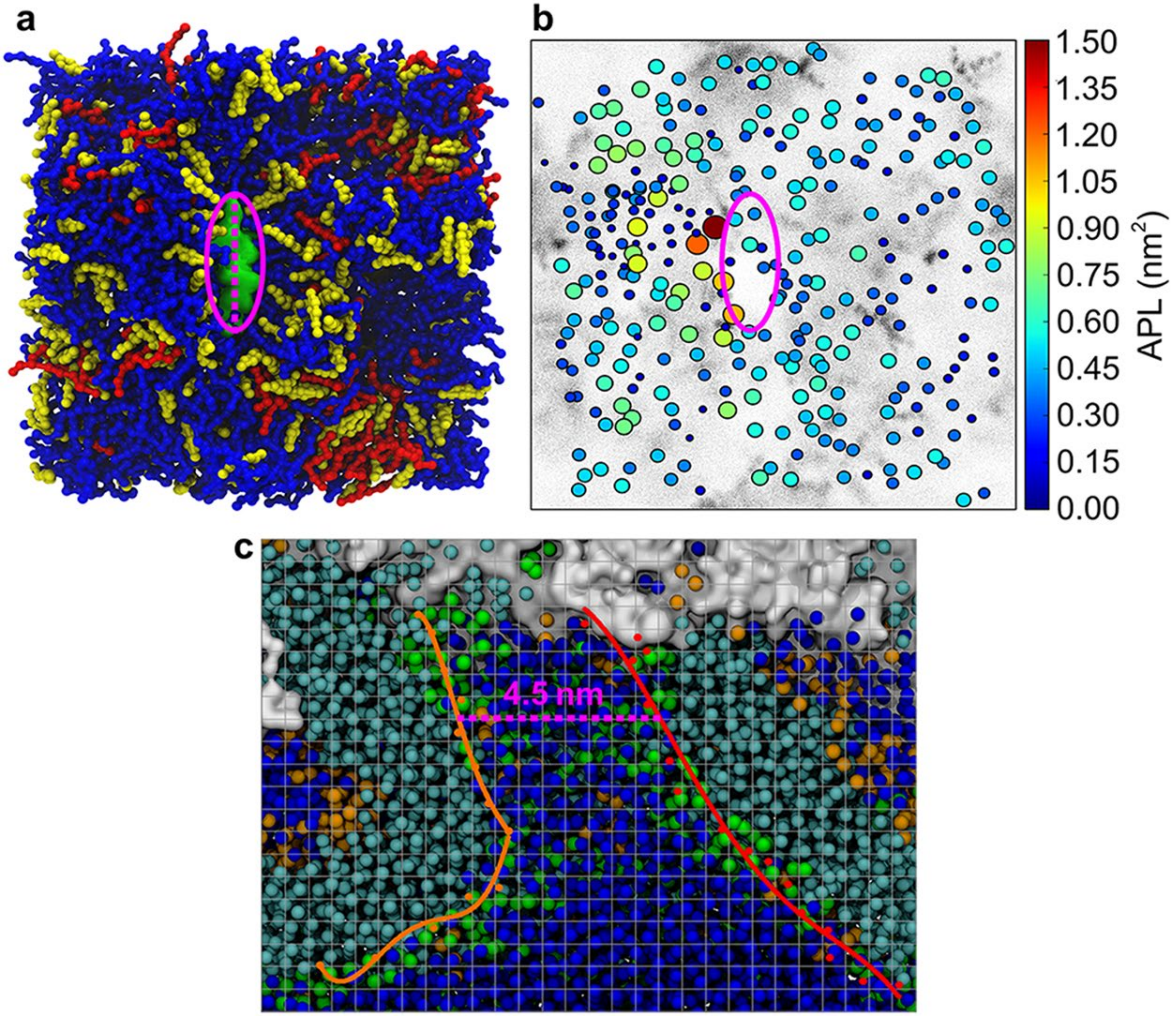
Properties of the membrane that lead to membrane poration. (**a**) Top view of the IN membrane at 100 µs. DPPC, DLiPC and CHOL are shown as CPK models in red, blue and yellow, respectively. The green surface and magenta oval are used to highlight the pore area in the membrane. The dashed line shows the diameter of the pore. (**b**) Scatter plot representing the positions of the lipid head beads of the outer and inner layers of the membrane. The size and color of each point in the plots correspond with the APLs of each head bead (see the color scale bar). The cloud in the background presents the density of CHOL. (**c**) Side view of the IN membrane. The atoms in the hydrophobic tails of the membrane are shown as cyan balls. The head groups of the lipid molecules in the outer and inner layers are shown in orange and green, respectively. The cluster of kB1 molecules is shown via a surface model in white. The water beads are shown as blue balls. The snapshot is overlaid by a scatter plot representing the position of lipid head beads that form the edge of the volcano-like channel. The plots are fitted with lines (shown with orange and red colors) to highlight the boundary of the channel. The average distance from the orange line to the red line is 4.5 nm. The size of each grid space is 0.5 × 0.5 nm.

In the second simulation condition of the IN membrane, we observed that two clusters of lipid molecules (in which CHOL and DLiPC were the majority) were extracted into two adjacent water-filled spaces inside the kB1 cluster^10^ (Fig. 4a). These clusters were connected by a bilayer area, called a “bilayer stalk” in this study. The density of the lipids in the stalk, particularly the CHOL molecules, was very low compared to the surrounding area (Fig. 5a and b). Therefore, the APLs of the lipid molecules in this area, especially in the inner layer due to the CHOL translocation, were remarkably high (>1.2 nm^2^/molecule) when compared to those in the surrounding area. Subsequently, permeation of water beads into the hydrophobic region of the bilayer stalk was detected (Fig. 4b). The lipids responded to this hydrophobic mismatch by pointing their polar heads to the permeating water. This rearrangement caused the fusion of lipid molecules between the outer and inner layers and resulted in the formation of a membrane pore (Fig. 4c). From the side view of the pore (Fig. 5c), the overall configuration of the pore channel was a volcano-like channel. The crater of the channel formed an elliptical shape where the diameter (major axis of the elliptic) was approximately 4.5 nm (Fig. 5a and c), which is comparable to a previous experimental study^9^. This crater was covered by kB1 molecules on the top. The diffusion of CG water beads across this kB1 cluster was observed to be limited^40^ (Fig. 4c). To investigate the ability of water molecules to move between the kB1 molecules that formed the cluster on the membrane surface, the CG model of this pored membrane system was reverse transformed to an atomistic (AA) model, and the simulation was conducted for 100 ns. In the AA model, the diffusion of water molecules inside the kB1 cluster as well as the formation of water wires (Fig. 4d) indicated that a water molecule moved along the water wire from the bottom to the top of the kB1 cluster. These results suggested that poration due to the binding of kB1 in the IN membrane is able to cause membrane leaking.

## Discussion

We constructed membrane models that contained DPPC, DLiPC, and CHOL to mimic the formation of lipid microdomains in HIV particle, HIV-infected cell, and host cell membrane models. Although these three lipids are not present in the real membranes, they were used in this study to facilitate the formation of the microdomain in the membrane models^36–38^. CHOL preferentially interacts with DPPC, which tightens the packing between the lipid molecules in the lo domain^41–43^. In contrast, DLiPC is loosely packed in the ld domain because of its polyunsaturated chains. The use of these lipids has been reported to promote phase separation in membrane models^23, 44^. As a consequence, the formation of the lipid microdomain was observed in all membrane models used in this study. The difference between these models is the amounts of cholesterol and sphingolipid (saturated lipid). The VI membrane contained the highest amounts of CHOL and DPPC, whereas IN contained the lowest amounts^27, 28^. Compared to the HO membrane, the lipid ratios imply that the virus gathers cholesterol and saturated lipid (sphingolipid) from the lipid microdomain of the host membrane to construct its envelope^25, 31^. We determined that the size of the lo domain in the VI membrane is the largest (VI ≫ HO > IN). The compressibility of the lipid molecules in the outer layer decreased from the center to the boundary of the lo domain (Supplementary Fig. S1). In contrast, the compressibility of lipid molecules in the inner layer in a corresponding area to the area of the lo domain in the outer layer was lower than the surrounding lipid molecules. Accordingly, we observed that lipid molecules tilt toward the center of the lo domain, i.e., they orient their head group to the domain center, forming a curved surface in each membrane (Supplementary Fig. S4). A curved surface was observed because the lo domain appeared only in the outer layer of each membrane model. The calculated curve radius (Supplementary Fig. S2) in the VI membrane was greater than that in the HO and IN membranes because the compactness of the VI membrane was greater than that of the IN and HO membranes, i.e., VI > HO ≈ IN. In contrast to the compactness, the surface area of the IN membrane was the largest, whereas the area of the VI membrane was the smallest, i.e., IN > HO > VI.

The roles of these physical properties, which were different between the membrane models, in the membrane disruption activity of kB1 were the focus of this study. Molecules of kB1 were desired to bind to the outer layers of our membranes because they contained lo domains only in these layers, which was based on the findings that cholesterol, sphingolipid, and saturated-lipids are enriched in this layer of the plasma membrane^29, 45, 46^. Our results show that, although the peptide reached the membranes at random locations, kB1 tended to be bound to the ld domain (Fig. 2). In the IN or HO membranes, the peptide could immediately bind to the ld domain because the size of the lo domain was small. In the VI membrane, which contained a bigger lo domain, some of the kB1 molecules bound to the lo domain while most of the molecules bound to the domain boundary. There was a report that indicated that it is more energetically favorable for peptides to move from the lo domain to the ld domain than to perturb the molecular packing of lipids in the lo domain^36^. However, in this simulation of the VI membrane, kB1 did not move from the lo domain to the ld domain. Instead, the DLiPC were observed to move into the lo domain. The lateral movement of kB1 is likely limited due to the specific configuration of the kB1 clusters formed in the simulation.

We also observed a clustering of CHOL with kB1 molecules that bound to the membrane. The basic functional group of Arg has been reported to interact with the hydroxyl groups of cholesterol, whereas the aromatic rings and aliphatic side chains of Trp, and Leu or Val interact with the hydrocarbon chain of cholesterol^47^. In this study, these residues of kB1 were absorbed into the interfacial zone of the membrane^11^, suggesting a preferential interaction between kB1 and CHOL. Because the kB1 molecules disfavor the lo domain, CHOL was determined to be sequestered from the lo domain by the kB1 molecules, which led to the lateral disorganization of the lo domain. The process occurred from the lo-ld domain interface to the center of the lo domain. The lo domain was quickly disorganized in the ordering of the IN > HO > VI membranes, indicating the direct involvement of the lo domain size. Simultaneously, the binding of kB1 caused the translocation of CHOL from the inner layer to the outer layer, which occurred at a higher rate in the IN membrane because cholesterol easily moves between the unsaturated bilayer^41, 43^. Nevertheless, the number of translocated molecules in the VI membrane was greater than the HO membrane. Possibly, it is more difficult for kB1 that is bound to the ld domain to sequester CHOL from larger sized lo domains than to extract CHOL from the inner layer. The translocation of CHOL reduced the compactness of the inner layer compared to the outer layer, which played a role in the poration process. The reducing rates in descending order are IN > VI > HO. The results revealed an interplay between the formation of the lipid microdomain and the membrane-binding behavior of kB1.

The asymmetric absorption of kB1 into the interfacial zone also caused deformation in the outer layer, which eventually led to membrane perturbation^10, 48^. The perturbation was mainly identified in the ld domain because less energy is required to compress and bend the bilayer in this domain^49, 50^. The perturbation was strongly related to the formation of the kB1 ring-like hollow^10^. In the large sized ld domain of the IN membrane, kB1 easily moved to form the hollow. However, crowding in the small ld domain increased the possibility of kB1 interacting and forming the hollow in the VI membrane. The distance amplitudes in descending order are IN ≈ VI ≫ HO. The amplitudes also indicated that the development of perturbation in the outer layer induced perturbation in the inner layer. The lipid molecules in the third amplitude level in the perturbed surface of the inner layer were identified in a small peak at a position corresponding to the edge of the kB1 ring on the outer layer. It is likely that the lipid molecules in the peak area moved vertically to compensate for the expansion of the lipid molecules in the outer layer due to the interfacial absorption of kB1. Combined with the compactness loss, the peak in the inner layer became the defect area, particularly in the IN membrane where the translocation of DLiPC in this area was observed (Supplementary Fig. S8b). Overall, the differences in the lipid ratios played a role in the response of the membranes to the binding of kB1. The membranes with less or more amounts of CHOL and DPPC (i.e., the IN or VI membrane), were more sensitive to kB1 binding than the HO membrane.

Second, we examined the roles of the membrane properties in the binding of kB1. The number of bound kB1 was highest in the IN membrane, followed by the HO and VI membranes, respectively. The reason for this was that the surfaces of the first two membranes were larger and flatter than the last membrane. In the VI membrane, kB1 molecules bound to the upper area of the curved surface (in the kB1-free state (Fig. 1)), which was occupied by the ld domain. Therefore, the kB1 molecules were localized in this small area of the ld domain because they preferred the ld domain more than the large sized lo domain. These findings indicate that the small surface area and high amplitudes of the curved surface in the VI membrane limited the binding of kB1. Regarding these results, we conclude that in addition to the effect of lipid composition^8, 17^, the differences in the lipid ratios also played a role in the binding of kB1.

Because the distance amplitudes were found to depend on the amount of kB1 that bounds to the membrane^10^, the amplitudes in the second condition of the HO membrane increased, whereas they slightly decreased in the VI membrane, compared to the first condition. However, the amplitudes in the VI membrane remained higher than those in the HO membrane. The amplitudes in descending order are IN > VI > HO. Therefore, the formation of differently sized lo domains indirectly affected the membrane disruption levels by regulating the number of kB1 that bound to the membrane.

Upon binding, kB1 disorganized the structure of the lo domain (Figs 2 and 5a) and caused membrane perturbation (Figs 3 and 4a). Moreover, in the second simulation condition, the perturbation led to the extraction of lipids into the water-filled space inside the kB1 clusters. Additionally, the poration that was observed in the IN membrane was initiated in the area of the bilayer stalk that connected the two clusters of lipid molecules extracted by kB1 (Fig. 4a). The APLs of the lipids in this area were remarkably high because lipids, especially CHOL, were extracted (Fig. 5b). It is likely that this reduction in the membrane compressibility enabled the water beads to permeate the bilayer stalk, which led to the fusion between the outer and inner layers (Fig. 4b). This mechanism is similar to the mechanism of cell fusion^51^ but, in our study, the bilayer fusion caused pore formation in the IN membrane. The configuration of the pore was similar to a volcano channel and, hence, called a volcano-like channel (Fig. 5c). The diameter of the volcano crater was comparable with the findings of a previous experimental study^9^. The crater was covered by kB1 molecules on the top and, because of the weak electrostatic potential of the CG water beads^40^, the movement of water beads inside this kB1 cluster was limited. When the CG model was transformed to an AA model, water molecules flowed inside the kB1 cluster from the inner layer side to the outer layer side, which reflected the leaking of the cell membrane (Fig. 4d). In the 100 µs of each simulation in the second condition, poration was not identified in the HO and VI membranes, although similar stalk formation as in the IN membrane was observed in each of them. This occurrence is possibly because the degrees of perturbation in these membranes were lower than that in the IN membrane (Table 1). However, our findings indicate that the VI membrane is more sensitive to the binding of kB1 than the HO membrane.

Our results show that the mechanism of membrane disruption by kB1 against different membrane types is very similar and corresponds with our previous simulations^10, 11^. The differences in the disruption levels among all of the membrane types were discussed based on this similar mechanism, and they were observed to relate to the different lipid ratios in the different membrane models. We note that poration in a volcano-like channel configuration was observed only in 100 μs in the IN membrane simulation system. The longer simulation time may reveal configuration changes of this membrane pore. Therefore, our results can be considered to be the initial state of the poration mechanism induced by the binding of kB1 to the interfacial zone of the membrane. Another important issue is the size of the simulation system. The simulation of a system containing a large number of molecules (in this case, the number of kB1 molecules) may have errors due to the interactions that the molecules may possibly experience across a periodic boundary. In this regard, our conclusions were made based on the trends of the membrane changes observed from the simulations of the systems containing lower numbers of kB1 (the first simulation condition) and based on the results comparing all of the membrane types.

## Conclusion

**Figure 6:**
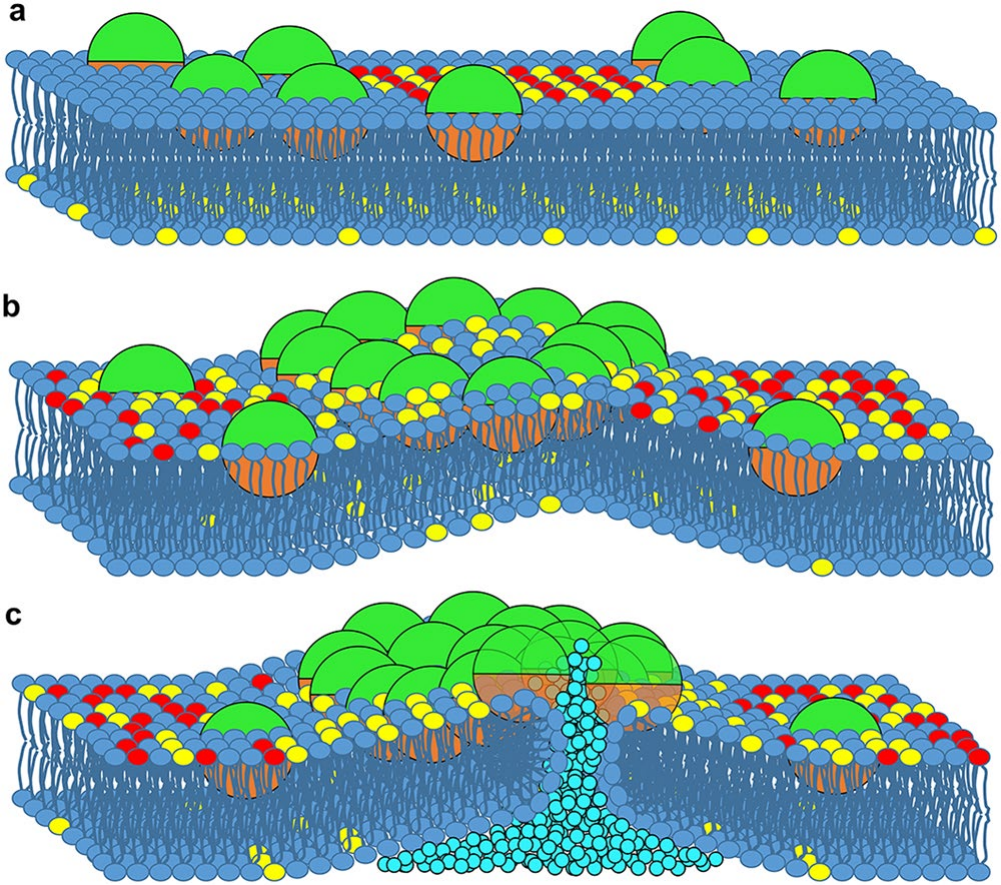
Overview of the membrane disruption mechanism of kB1. (**a**) The hydrophobic surface (orange) initiates and stabilizes the binding of kB1 to the membrane, while the hydrophilic surface (green) interacts with the lipid polar heads and water. The kB1 molecules prefer binding to the ld domain. (**b**) The asymmetric membrane binding of kB1 induces lipid lifting. The lipids are lifted the highest inside the kB1 ring-like hollow. The deformation in the outer layer consequently induces lifting of the lipids in the inner layer. The kB1 molecules disorganize the lo domain and cause the translocation of CHOL from the inner layer to the outer layer. (**c**) The perturbation and extraction make a defect area where lipid molecules are loosely packed, which allows the permeation of water molecules (light blue color) into the bilayer. The fusion between the outer and inner layers responding to the permeation of water eventually causes the membrane pore to form a volcano-like shape. The water molecules leak from the inner layer side to the outer layer side by diffusing inside the kB1 cluster that covers the volcano crater. The head groups of DPPC, DLiPC, and CHOL are shown in red, blue and yellow, respectively.

We investigated several aspects of the interplay between the amounts of lipids in the VI, IN and HO membranes and the membrane disruption activity of kB1. First, the differences in the amount of lipid affect the amount of kB1 that will bind to the membrane as they determine the size of the lo domain and, hence, the surface area of the membrane-landing area for kB1. Second, the membrane that contained low amounts of CHOL and DPPC (the IN membrane) was found to be easily perturbed by kB1 because its lipid molecules were loosely packed. However, the membrane that contained high amounts of CHOL and DPPC (the VI membrane) was promptly deformed because of the highly compressed lipids in its large sized lo domain. Moreover, the large sized lo domain caused the crowding of kB1 in a small area of the ld domain. The crowding of interfacially absorbed peptide molecules has been reported to be one mechanism that induces membrane curvature^48, 52^. Finally, the lipid ratio differences were observed to be relevant to pore formation. The poration in the IN membrane was initiated when water permeated the inner layer. CHOL translocation was observed to be one factor that affected the water permeation. The translocation was found to occur quickly in the IN membrane that contained loosely pack lipids. These properties caused the disruption activity of kB1 against the VI, IN and HO membranes to be of unequal magnitude. We also note that, although poration was not detected in 100 μs for the VI membrane simulation, kB1 may inhibit infection with HIV by rapidly covering the small surface area of the viral membrane. Moreover, the disorganization of the lo domain may inhibit fusion between HIV and host cell membranes because both cholesterol and the lipid microdomain are critical for fusion^25, 31^. Furthermore, our results indicate that the VI membrane is more sensitive to kB1 binding than the HO membrane, suggesting that kB1 activity is more effective in the VI membrane. Finally, based on results from this and our previous studies^10, 11^, we present an updated model of the membrane disruption mechanism of kB1 in Fig. 6.

## Methods

### Simulation details

The GROMACS simulation package^53, 54^ version 4.5 was used to conduct all MD simulations in this study. The Martini force field, version 2.1^32–34^ was used in the CG MD simulations, and GROMOS53a6^55^ was used in the AA simulations. Lipid force fields were obtained from the Lipidbook^56^ database. The CG model of the IN membrane system at 100 µs in the second simulation condition was reverse-transformed to the AA models using the method developed by Wassenaar *et al*.^57^. All MD simulations were performed under the NPT ensemble. The temperature was independently coupled to 310 K using a Berendsen thermostat for each molecule type in the system^58^. The pressure was coupled to 1 atm with a compressibility of 4.5 × 10^−5^ bar^−1^ using a Berendsen barostat^58^ with a semi-isotropic scheme to obtain a tensionless membrane. A periodic boundary condition with standard non-bonded interaction criteria was used. A leapfrog integration algorithm with a time step of 20 fs was used to solve the motion equation. The van der Waals and electrostatic interaction cut-offs were set to 1.2 nm. Visual molecular dynamics (VMD) software^59^ was used to visualize the simulation results. The data was analyzed separately for the inner and outer layers. We used the Leaflet Finder algorithm in the MDAnalysis toolkit^60^ to identify the phospholipids in each layer.

### Preparation of a CG model of kB1

Similar to our previous study^10, 11^, two computational scripts, including atom2cg_v2.1.awk and seq2itp.pl were downloaded from the Martini web site (http://md.chem.rug.nl/cgmartini/images/tools) to prepare the CG model of kB1. The first script was used to translate the atomic coordinates of the kB1 solution structure, which was obtained from the protein data bank (PDB code: 1NB1^61^), to CG coordinates. In the CG model, a set of heavy atoms (frequently four atoms) is mapped onto a pseudo-atom^33^. This reduction in the degree of freedom enables the CG model to be used to simulate the complex processes of peptide-membrane interactions^62^. The second script was used to generate topology files that could be interpreted by the GROMACS simulation package. This file linked to the intra- and interaction parameters in the Martini force field file. We adapted the topology file slightly to define the peptide bond between the N and C termini in the cyclic structure of kB1. Based on the kB1 sequence, the parameters of the peptide bond between Cys1 (the C-terminus) and Val29 (the N-terminus) were obtained from the bond parameters between Val21 and Cys22. Standard protonation states at neutral pH were assigned to all amino acid residues, which resulted in a total charge of zero.

### Preparation of the membrane model

The membrane model was prepared according to the protocol in ref. 10. Each membrane model was constructed using in-house software in which the lipid molecules were aligned to form bilayers. The program automatically generated the lo domains in the outer layer by clustering DPPC with CHOL at a ratio of 1:1 in relation to their molecular shape matching^20^. The distribution of CHOL was set to be equal in the outer and inner layers. There were 1,000 lipid molecules for each membrane (Supplementary Table S1). The DPPC:DLiPC:CHOL ratios were translated from the lipid ratios obtained in an experimental study^28^. Each lipid molecule was hydrated with 6 water beads^63^. For each model, a simulation of 4 µs was conducted to relax the interaction between the lipid molecules.

### Simulation system setup

Systems of each membrane model at 20 µs were used to setup a system to assess the kB1 activity. Molecules of kB1 were added to each membrane system using two different protocols. In the first condition, the kB1 molecules were continuously added to each membrane system every 20 µs by replacing water molecules similar to the procedure used in ref. 10. The concentration steps were 24, 48, 72, 96, and 120 kB1 molecules/1,000 lipid molecules/24,000 water beads. Using this protocol, kB1 molecules bound to the membrane when they were in monomeric or small oligomeric form. The simulation duration for each concentration step enabled the peptide to translocate from an oligomer to the membrane surface^10^. Therefore, most kB1 molecules could bind with the membrane. In each concentration step, the membrane type system was continued to the next concentration step only when the number of kB1 molecules bound to it was almost equal to the number of kB1 in the other two membranes. If the numbers were very different, the process was repeated for that concentration step. Water molecules were subsequently added such that each kB1 concentration met the desired amount of water molecules; i.e., 24,000 CG water beads corresponding to 96,000 molecules of AA water. In the second condition, the center of mass of the membrane was translated to be located approximately 10–20% lower than the center of the simulation box in the Z axis. Three hundred fifty molecules of kB1 were subsequently added to each membrane system at once. Using this protocol, the size of the system was smaller than that of the simulation that placed the membrane at the center of the simulation box^10^. Because the charge distribution on the surface of kB1 is asymmetrical, the molecules of the peptides were added to the system with a random geometry orientation using the Genbox program.

### Data analysis

We implemented a Python script based on the MDAnalysis toolkit^60^ to analyze the MD simulation results. To analyze the distance amplitude, the Cartesian coordinates of the selected lipid head bead (PO4 bead^32^) of each phospholipid molecule in each layer were extracted. The coordinates were sorted using the Z axis values, and the minimum value was used as a reference value. The value of each head bead was subtracted from the reference value to obtain its amplitude.

The interlayer CHOL distribution was analyzed by counting the number of CHOL molecules within 1.2 nm around the lipid head bead of each phospholipid molecule in each layer. The redundant selection of CHOL within the cutoff was checked, confirming that the number of CHOL molecules in the layer was not incremented. The CHOL molecules outside of the cutoff were considered to be located between the layers.

The area per lipid head bead was measured using the g_lomepro program, which was designed to analyze the properties of local membranes^64^. The program uses a GridMAT-MD algorithm^65^ in which the area of each head bead is calculated after all head beads are mapped onto the grid space assigned by the algorithm.

To measure the clustering of the lipid molecules, the DBSCAN algorithm^39^ was applied. The Cartesian coordinates of the lipid head beads were sent to the DBSCAN class obtained from Scikit-learn^66^. The distance cutoff and the minimum numbers of head beads in the cluster were set to 1.5 nm and 3 atoms, respectively^67^.

The solvent accessible surface area (SASA) was estimated using a GROMACS utility program (g_sas) with 0.56 nm of the solvent probe radius^68^. The calculated SASA was used to present the surface area of the membrane and the lipid microdomain.

## Electronic supplementary material

Supplementary Information
