# Supplementary material for: Molecular dynamics exploration of poration and leaking caused by Kalata B1 in HIV-infected cell membrane compared to host and HIV membranes
Source: Sci Rep. 2017 Jun 15;7:3638. doi: 10.1038/s41598-017-03745-2 (PMC5472625; doi:10.1038/s41598-017-03745-2)
Supplement: Supplementary file 1 — Supplementary Information [file 41598_2017_3745_MOESM1_ESM.doc]

Supplementary

**Molecular dynamics exploration of poration and leaking caused by Kalata B1 in HIV-infected cell membrane compared to host and HIV membranes**

Wanapinun Nawae^1^, Supa Hannongbua^2^ and Marasri Ruengjitchatchawalya^3,4 *^

^1^Pilot Plant Development and Training Institution, King Mongkut’s University of Technology Thonburi (Bang Khun Thian Campus), 49 Soi Thian Thale 25, Bang Khun Thian Chai Thale Rd., Tha Kham, Bang Khun Thian, Bangkok 10150, Thailand. E-mail: [wa_nawae@tu.ac.th](mailto:wa_nawae@tu.ac.th)

^2^Department of Chemistry, Kasetsart University, 50 Phaholyothin Rd., Ladyao Chatuchak, Bangkok, Thailand, 10900. E-mail: fscisph@ku.ac.th

^3^Biotechnology program, School of Bioresources and Technology, King Mongkut’s University of Technology Thonburi (Bang Khun Thian Campus), 49 Soi Thian Thale 25, Bang Khun Thian Chai Thale Rd., Tha Kham, Bang Khun Thian, Bangkok 10150, Thailand.

^4^Bioinformatics and Systems Biology Program, King Mongkut’s University of Technology Thonburi (Bang Khun Thian Campus), 49 Soi Thian Thale 25, Bang Khun Thian Chai Thale Rd., Tha Kham, Bang Khun Thian, Bangkok 10150, Thailand. E-mail: marasri.rue@kmutt.ac.th, Telephone: +66-2470-7481

Supplementary figures


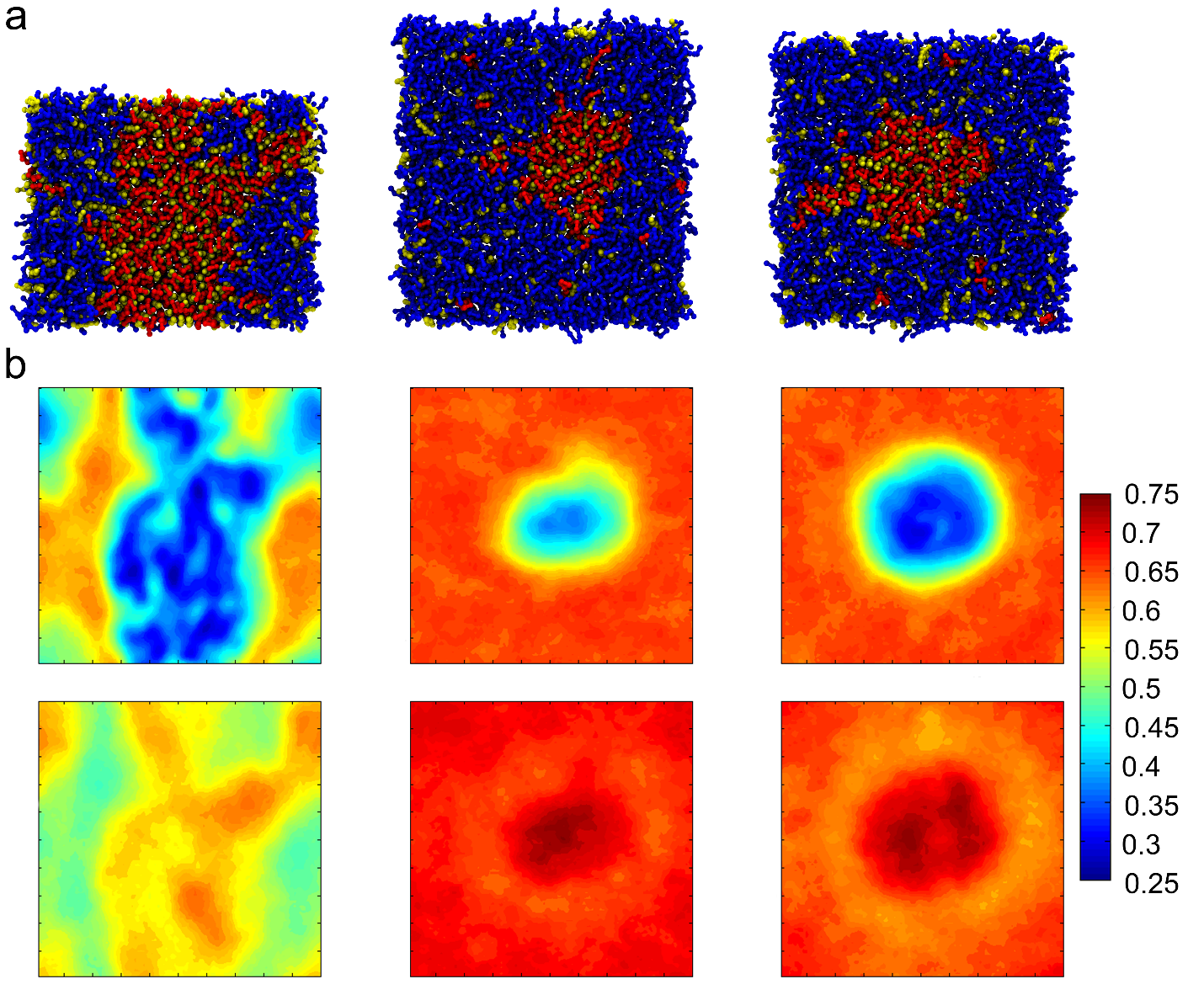


**Figure S1.** Average area per lipid. (a) The top view of the HIV (VI), HIV-infected (IN) and uninfected host (HO) membrane models are shown from left to right. DPPC, DLiPC and CHOL are shown as a CPK model with red, blue and yellow colors, respectively. (b) Heat maps display the average area per lipid (APLs) in the different local areas of the membrane models are shown from left to right. The APLs of the outer layer are shown in the upper panel while those of the inner layer are shown in the lower panel. The APLs shown in the scale bar are presented in unit of nm^2^.


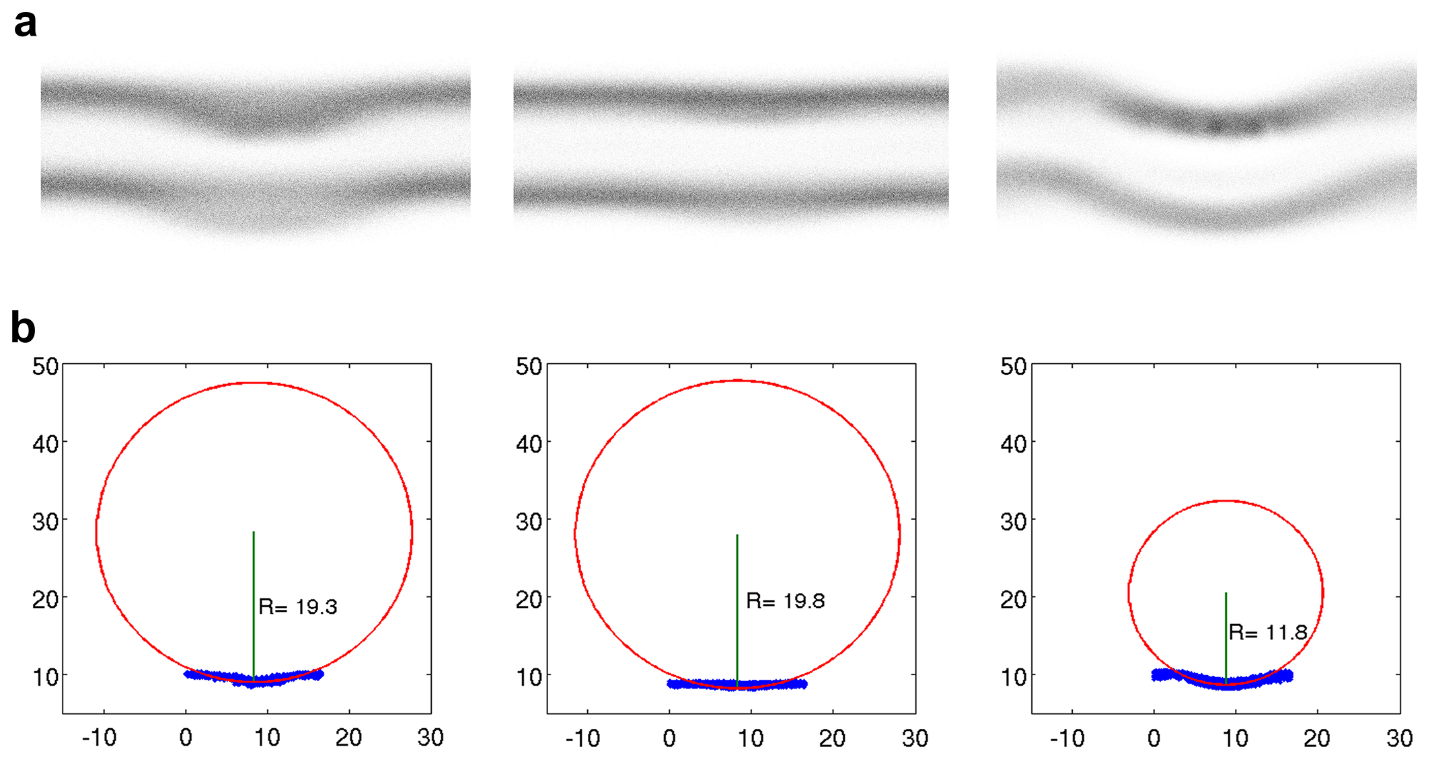


**Figure S2.** Measurement of curved surface radius. (a) Density plot of lipid head beads is plot for both layer of each membrane from its side view. From left to right, the densities for the HO, IN and VI membrane are shown. The X and Y coordinates of each density dot are extracted for the outer layer of each membrane. (b) To measure radius of curved surface, least squares circle fitting is applied to each set of density dots using MATLAB programming script. The radius R is shown in angstrom (Å) unit.

**
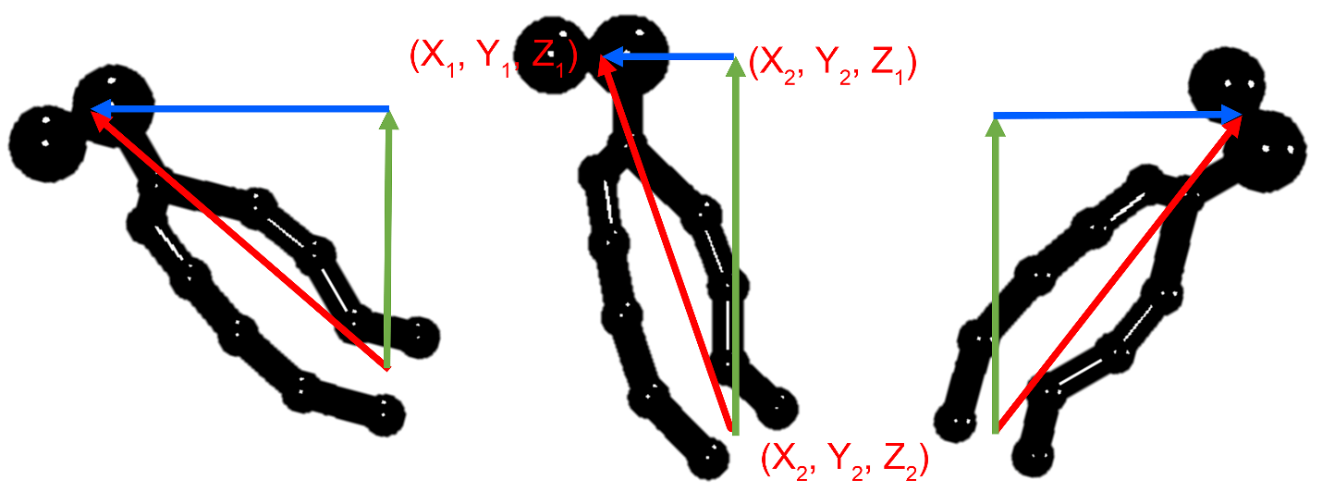
**

**Figure S3.** Analysis of lipid tilt angle. To calculate tilt angle of each lipid molecule the Cartesian coordinate denoted as (X_1_, Y_1_, Z_1_) and (X_2_, Y_2_, Z_2_) corresponding to position of the COM of the head atoms and tail atoms of each lipid molecule are identified. The red vector is calculated to define major axis of the lipid molecule. The green vector is calculated to represent normal axis of the membrane. The tilt angle is the angle between green and red vectors. The blue vector (draw from the tip of the green vector (X_2_, Y_2_, Z_1_) to the tip of the red vector (X_1_, Y_1_, Z_1_)) is used to represent tilt angle where the vector length corresponds to the angle and the vector direction corresponds to the tilt direction of each lipid molecule.


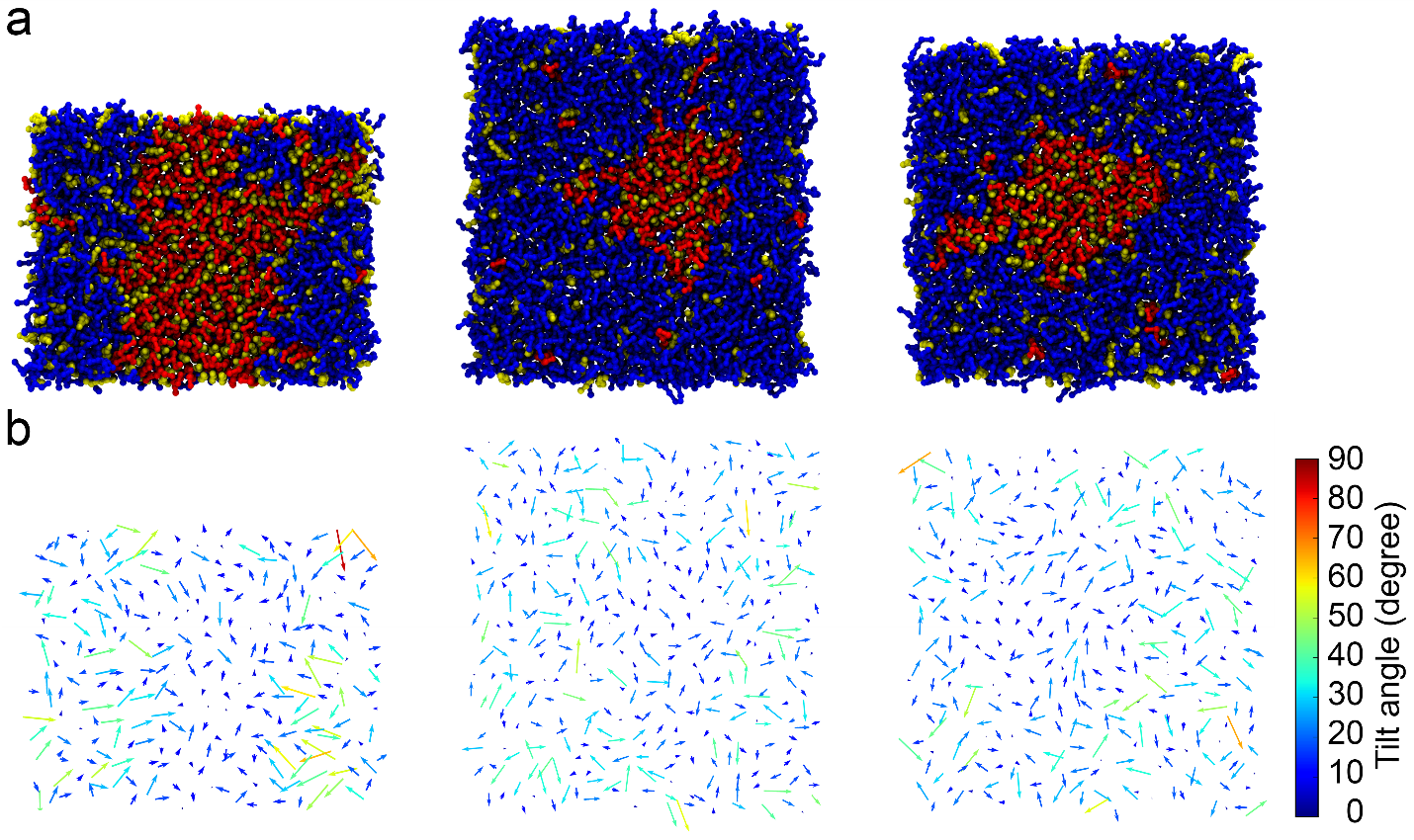


**Figure S4.** Tilting of lipid molecules toward the lo domain. (a) The top view of the VI, IN and HO membrane models are shown from left to right. DPPC, DLiPC and CHOL are shown as a CPK model with red, blue and yellow colors, respectively. (b) The vectors represent lilt angle of lipid molecules in the outer layer of the VI, IN and HO membrane models are shown from left to right. Length and color of each vector corresponding to tilt angle (see color scale bar). Direction of the vector corresponds to the tilt direction of each lipid molecule.


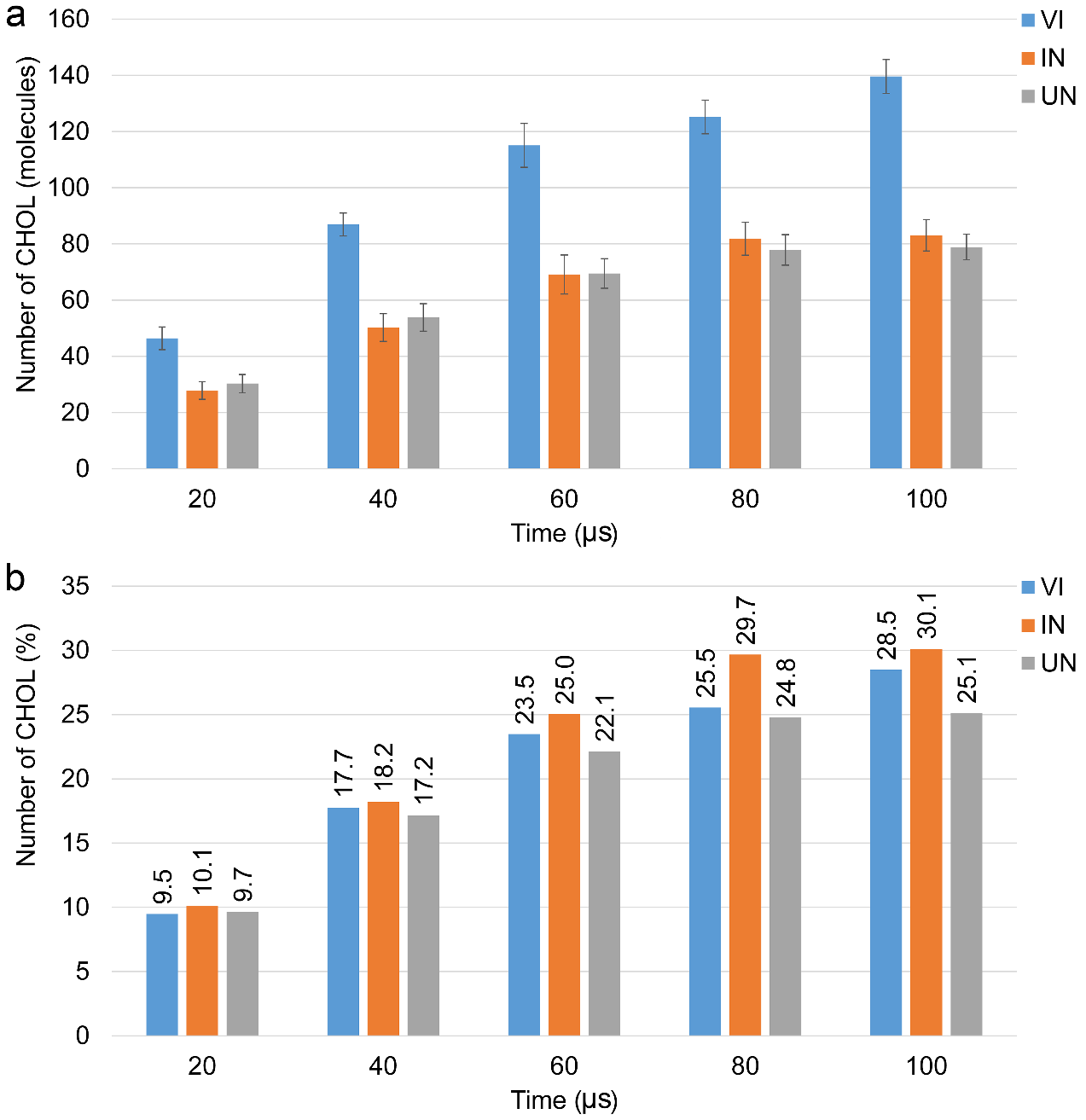


**Figure S5.** Size of the CHOL cluster surrounding membrane-bound kB1 molecules. (a) Amount of CHOL molecules locate within 1.2 nm of the kB1 molecules that bound with each membrane. Error bars shown standard deviation of the amount of CHOL molecules that was averaged every 20 µs for each layer of each membrane. (b) The amount of CHOL molecules is shown as percentage to total CHOL molecules in each membrane system (see Table S1). The number of kB1 molecules is increased every 20 µs (see Table 2).


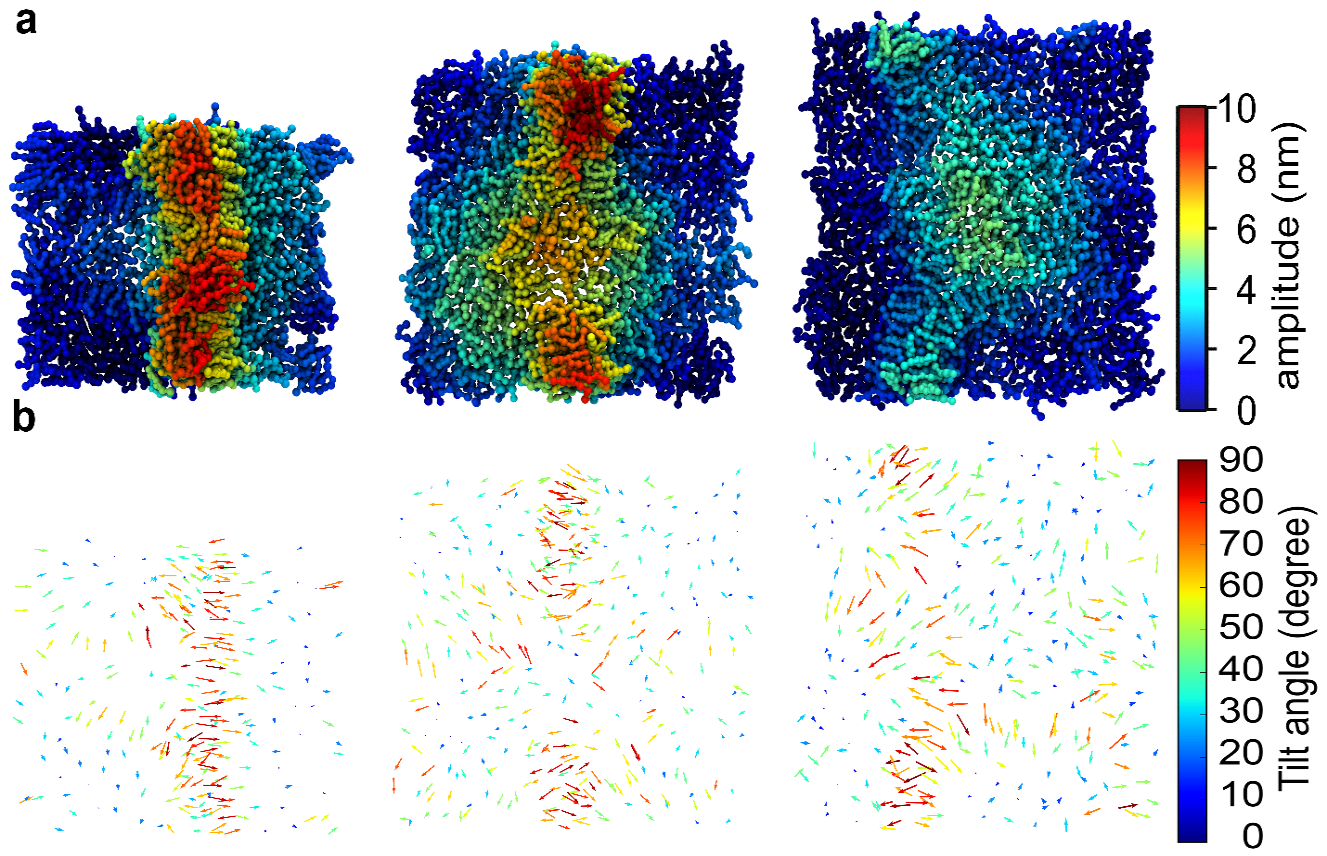


**Figure S6.** Tilting of lipid molecules in the folding area of the membranes. (a) The top view of the VI, IN and HO membrane models at 100 µs are shown from left to right. Lipid molecules are shown as a CPK model. The color of each lipid molecule represents its distance amplitude (see color scale bar). The kB1 molecules are not shown. (b) The vectors represent lilt angle of lipid molecules in the outer layer of the VI, IN and HO membrane models are shown from left to right. Length and color of each vector corresponding to tilt angle (see color scale bar). Direction of the vector corresponds to the tilt direction of each lipid molecule.


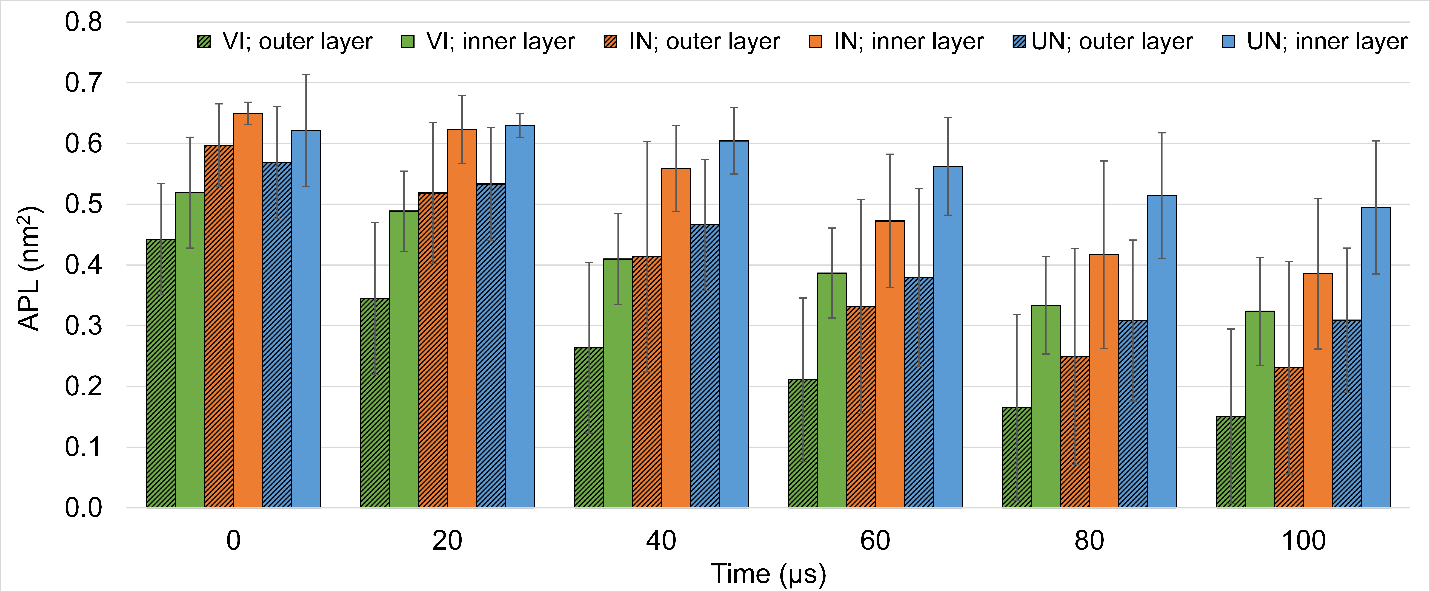


**Figure S7.** Compactness change of the membrane regarding the number of membrane-bound kB1 molecules. The area per lipid averaged over all lipid molecules (including in those in the lo and ld domains) in each layer of VI, IN and HO membrane models are shown. The number of kB1 molecules is increased every 20 µs (see Table 2). Error bars shown standard deviation of the averaged APL values.


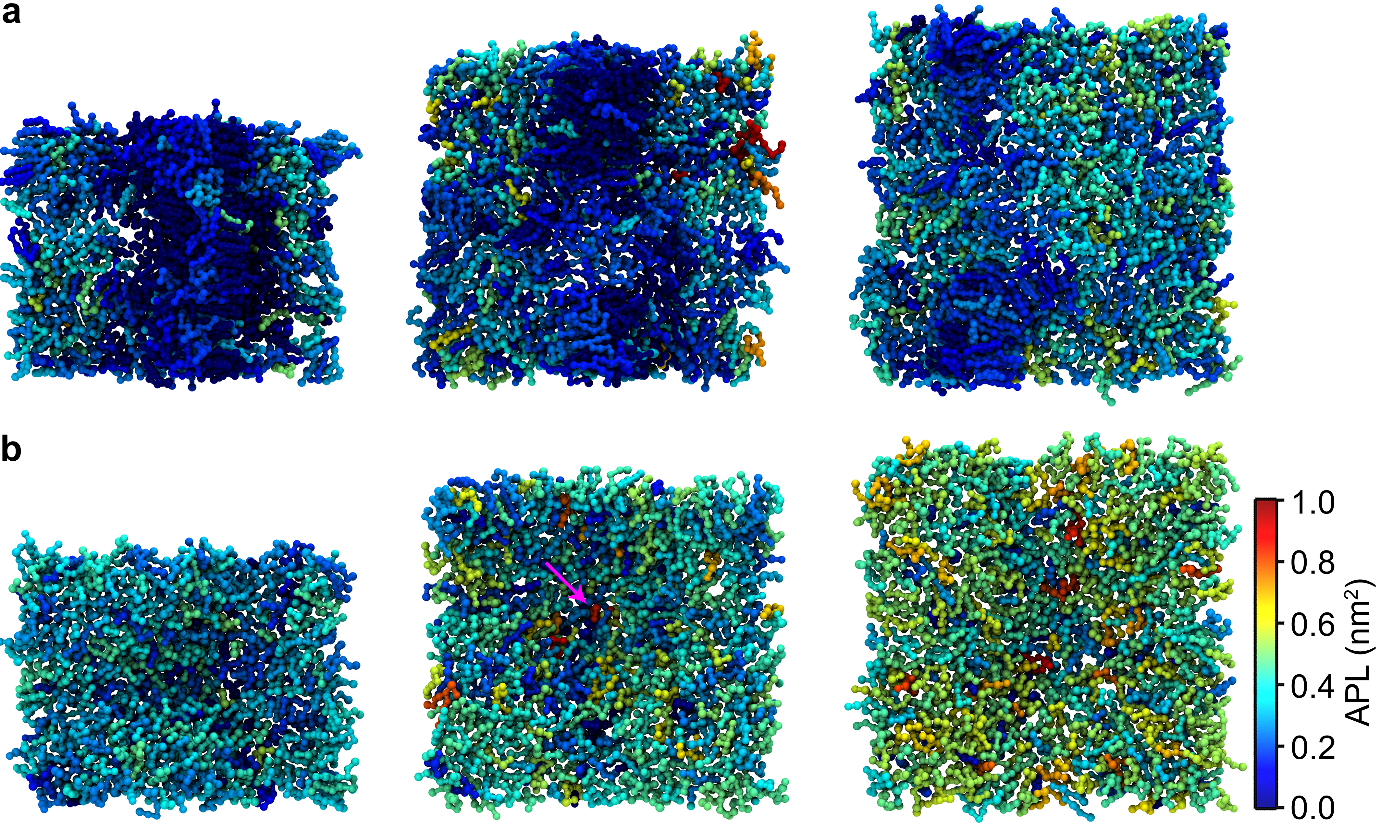


**Figure S8.** Area per lipid molecule. (a) Top view of the outer layer of the VI, IN and HO membranes at 100 µs are shown from left to right. (b) Bottom view of the inner layers of the membranes are shown. Lipid molecules are shown as a CPK model. The color of each lipid molecule is represented its APL value (see color scale bar). The magenta arrow points the DLiPC molecule that translocate from the outer layer to the inner layer in the IN membrane. Positions of each lipid molecule in each membrane corresponding to that shown in Figure 3.

Supplementary table

**Table S1. Composition and amount of lipid molecules in each membrane**

| Membrane | Composition of lipid molecules | | |
| --- | --- | --- | --- |
|  | DPPC | DLiPC | CHOL |
| HIV | 127 | 383 | 490 |
| HIV-Infected | 58 | 667 | 276 |
| Uninfected | 67 | 620 | 314 |

**Table S2.** Amount of CHOL molecules in the outer, inner and between layers of the VI, IN and HO membrane models. A CHOL molecules is consider to located in the outer or inner layer if the distance from its head atom to the head atom of any phospholipid molecules in that layer is within 1.2 nm. A CHOL molecules is consider to orient between the layers if the distance is greater than 1.2 nm

| Time (µs) | Amount of CHOL molecules | | | | | | | | |
| --- | --- | --- | --- | --- | --- | --- | --- | --- | --- |
|  | VI | | | IN | | | HO | | |
|  | outer | inner | between | outer | inner | between | outer | inner | between |
| 0 | 269 | 188 | 33 | 133 | 121 | 22 | 165 | 113 | 36 |
| 20 | 302 | 165 | 23 | 140 | 115 | 21 | 176 | 119 | 19 |
| 40 | 318 | 143 | 29 | 171 | 85 | 20 | 179 | 118 | 17 |
| 60 | 350 | 119 | 21 | 184 | 75 | 17 | 196 | 106 | 12 |
| 80 | 361 | 110 | 19 | 200 | 63 | 13 | 196 | 97 | 21 |
| 100 | 373 | 101 | 16 | 202 | 63 | 11 | 203 | 95 | 16 |
